# Lessons learned from a community based intervention to improve injection safety in Pakistan

**DOI:** 10.1186/1756-0500-6-159

**Published:** 2013-04-22

**Authors:** Arshad Altaf, Sharaf Ali Shah, Kulsoom Shaikh, Fiona M Constable, Selma Khamassi

**Affiliations:** 1 Bridge Consultants Foundation, 4-E, Block-6, PECHS, Off Sharahe-e-Faisal, Karachi 74500, Pakistan; 2 World Health Organization, Avenue Appia 20, Geneva 1211 27, Switzerland

**Keywords:** Unsafe injections, Community intervention, Rural Pakistan

## Abstract

**Background:**

A national study in 2007 revealed that in Pakistan the prevalence of hepatitis B is 2.5% and for hepatitis C it is 5%. Unsafe injections have been identified as one of the reasons for the spread of these infections. Trained and untrained providers routinely perform unsafe practices primarily for economic reasons i.e. they reuse injection equipment on several patients. The patients, do not question the provider about the need for an injection because of social barriers or whether the syringe is coming from a new sterile packet due to lack of knowledge. The present paper represents an intervention that was developed to empower the community to improve unsafe injection practices in rural Pakistan.

**Methods:**

In a rural district of Pakistan (Tando Allahyar, Sindh) with a population of approximately 630,000 a multipronged approach was used in 2010 (June to December) to improve injection safety. The focus of the intervention was the community, however providers were not precluded. The organization of interventions was also carefully planned. A baseline assessment (n=300) was conducted prior to the intervention. The interventions comprised large scale gatherings of the community (males and females) across the district. Smaller gatherings included teachers, imams of mosques and the training of trained and untrained healthcare providers. The Pakistan Television Network was used to broadcast messages recorded by prominent figures in the local language. The local FM channel and Sunday newspaper were also used to disseminate messages on injection safety. An end of project assessment was carried out in January 2012. The study was ethically reviewed and approved.

**Results:**

The interventions resulted in improving misconceptions about transmission of hepatitis B and C. In the baseline assessment (only 9%) of the respondents associated hepatitis B and C with unsafe injections which increased to 78% at the end of project study. In the baseline study 15% of the study participants reported that a new syringe was used for their most recent injection. The post-intervention findings showed an increase to 29% (n=87).

**Conclusion:**

It is difficult to assess the long-term impact of the intervention but there were several positive indicators. The duration of intervention is the key to achieving a meaningful impact. It has to be at least 18–24 months long.

## Background

Pakistan is a large country of approximately 176 million people (UNICEF). Due to multiple reasons related to the quality and availability of health services at public sector facilities, patients prefer to go to private practitioners. It is estimated that 70-80% of healthcare in Pakistan is provided by private practitioners and general practitioners (GPs-both trained and untrained) are at the front line of this provision. These GPs are also responsible for almost all unsafe injection practices which are quite common in Pakistan in rural and urban settings. The administration of injections in developing countries often leaves much to be desired. Many injections are given for the wrong indications such as acute respiratory infections, diarrhea, skin injections and urinary tract infections. In some countries children receive an alarmingly large number of injections [1-6]. Many studies during the past 15 years have shown that the reuse of injections transmits hepatitis B (HBV) and C (HCV) in Pakistan [7-16]. Studies have also reported a variable rate of injections provided with used syringes, ranging from 40% to 94% [8,11]. Using field-tested focus group guides from WHO, 18 focus group discussions (FGDs) were conducted with community members of rural Sindh, peri-urban and urban Karachi during January-February 2001. Results indicated that injections were overused in Sindh because patients preferred them, believing that they provided quick relief, and that they perceived them as a therapeutic norm and standard practice. However, according to the community, the initiative for prescribing an injection was taken by doctors [16]. The national study conducted by the Ministry of Health (MOH) in 2007 indicated the overall prevalence of HBV to be 2.5% and HCV 5%. A strong association between reuse of injection equipment and disease transmission was once again established [17].

In 2010, the MOH conducted a mapping of injection providers in Rawalpindi (Punjab province) and Tando Allah Yar (Sindh province) districts to estimate their numbers [18]. The population of Tando Allah Yar (TAY) is 630,000 and is located about 200 kilometers northeast of Karachi which is the provincial capital and trading hub of the country. In TAY 783 injection providers were identified and among them 55% were dispensers (untrained), GPs 25%, homeopaths 17%, hakims 2% and others 4%. All of these providers were prescribing injections (18).

The providers have two key motives for prescribing unnecessary injections. The first is economic, as a prescription with an injection costs more compared to one that does not have an injection included in it [16]. Second, injections do provide quick relief [19]. The idea of getting an injection has become so rooted in uneducated communities that it requires a multipronged approach to improve knowledge and change practices. The present intervention was implemented between June and December 2011 in the Tando Allahyar district of Pakistan with the objective of improving unnecessary and unsafe injection practices using a multipronged approach.

## Methods

### Setting

Tando Allahyar is a rural district of the Sindh province of Pakistan with an approximate population of 630,000. There are three administrative units of the district called ‘taluka’ in the local language. The district has an agricultural economy and the main products include mangoes, guavas, cotton, wheat and sugar cane on a large scale. However, there is economic disparity and poverty is widespread. The literacy level in the district is also low as documented in a previous study. Sindhi is the predominant ethnicity and language spoken in the district followed by Urdu.

### Interventions

A multipronged approach was used in designing interventions. The focus of the interventions was the community, however providers were not precluded. The course of interventions was also carefully organized. As a first step after consulting the community, it was decided to organize large scale gatherings for 200–300 people and the timing of the event was also set by consensus for the early evening followed by dinner. In the large gatherings, health messages were delivered in Sindhi and Urdu languages along with videos in which prominent Sindhi personalities also gave injection safety messages. Following the large event, group meetings of 20-25 persons were organized targeting teachers (male and female) and religious leaders. Both these groups are respected in the rural communities. Educational material, with illustrations in the local language, was widely distributed.

Healthcare providers of the area, both qualified i.e. with a MBBS degree, and untrained, were also involved and half-day events were organized with these groups. In the sessions a half-hour talk on injection safety was given, followed by an interactive session focusing on rational use of injections and avoiding unsafe injection practices. Table 1 provides details of the community based events.

**Table 1:** Activities conducted in District Tando Allahyar (September-November 2011)

**Date**	**Activity/Target population**	**Venue**	**Number of participants**
12^th^ September	Large community gathering attending by adult males and mothers	Chambur	450-500
15^th^ September	Teachers (female)	Venus School Tando Allahyar	10-15
18^th^ September	Teachers (Male)	Main Girls Primary School Tando Allahyar	10-15
22^th^ September	Teachers (Female)	SM College Tando Allahyar	10-15
29^th^ September	Imams	Bridge Field Office Tando Allahyar	10-15
30^th^ September	Teachers (Male)	Darul-ulom High School Tando Allahyar	10-15
10^th^ October	Imams	Bridge Field Office Tando Allahyar	10-15
19^th^ October	Healthcare providers (trained and untrained)	Pakistan Medical Association House-Tando Allahyar	54
27^th^ October	Healthcare providers	Usman Shah Hori	20-25
27^th^ October	Large community gathering attending by adult males and mothers	Jhando Mari	200-250
29^th^ October	Teachers (male and females)	Piyaro Lund	20-25
4^th^ November	Healthcare providers	Chamber	20-25
7^th^ November	Teachers	Bukera Sharif	20-25
11^th^ November	Large community gathering attending by adult males and mothers	Misan Wadi	250-300
11^th^ November	Imams	Misan Wadi	15-20
25^th^ November	Father and priests	Main Church Tando Allahyar	5
27^th^ November	Pandits	Main Mandir Tando Allahyar	4

In October 2011 a local radio channel was used to broadcast injection safety messages in the Sindhi language for one week. Following that local cable operators were hired and they broadcast the same messages for one month around the clock including prime time.

A local FM channel was also used to broadcast injection safety messages and the most widely read Sindhi daily newspaper was used to print messages in the Sunday newspaper.

### Sample size for baseline and end of project assessment

Post-hoc power calculation estimated that sample sizes of 300 in pre intervention and after the intervention could achieve 86% power to detect a difference between the group proportions of 10%. The proportion in control group was assumed to be 15%. The significance level was 5%. The purpose of the baseline survey was to determine what kind of messages and means should be used to educate the community in Tando Allahyar about injection safety. An end of project study was done to assess the effect of interventions.

Both studies were conducted by trained community health workers. It was decided to conduct 100 interviews in each administrative unit and to select respondents from all social categories in order to get a wide cross-section of responses representative of the study district.

### Estimated population covered by the intervention

By using a multipronged approach we were able to include 20-25% of the population i.e. between 126,000 to 157,000 persons in Tando Allahyar district.

### Ethical review

The Ethical Review Committee of Bridge Consultants Foundation is registered and consists of highly qualified persons. The study was ethically reviewed and approved. Verbal consent was obtained from all respondents and the questionnaires were implemented in the Sindhi and Urdu languages. While piloting the questionnaire we explored the different options for types of consent. The participants were reluctant to provide written consent and were more comfortable with oral consent. The questionnaire had very few personal questions such as about age or education. The other questions were related to healthcare seeking behaviour and injection safety.

## Results

### Baseline findings

The majority of the participants (77%) were male. The average age of the participants was 36.2 years. The categories are detailed in Table 2.

**Table 2:** Details of participants in the baseline assessment

**Category**	**Percentage (n=300)**
Land owners	15%
Union Council elected representatives	8%
School teachers	17%
Social workers (NGO/CBO)	14%
Imams	8%
Taxi and rickshaw drivers	16%
Representatives of pharma companies	9%
Medical store owners	13%

About 38% (n=114) of the participants said that the decision to select a provider depended on the convenience/proximity of the clinic followed by the qualifications of the providers 35% (n=105) and then the experience of the providers 27% (n=81). Fever and common cold (41%) were the most frequently reported illnesses (123 respondents) followed by general aches and pains (23%, n=69) and hepatitis (12%, n=36).

Most of the participants 53% (n=159) considered that drinking dirty water was associated with hepatitis B and C, followed by hot (*garam*) food 20% (n=60). A small number of the study participants 9% (n=27) were aware that the reuse of syringes can cause hepatitis B and C.

The overwhelming majority of community participants 82% (n=249) considered that injections work faster than oral medicines, giving this as the reason for preferring them. A small proportion 18% (n=54) said that they leave the prescribing decision to the health care provider.

Approximately half of the participants 49% (n=147) reported that they had received an injection in the last month from a clinic or healthcare provider. Only 15% (n=45) of the respondents were aware whether the syringe was new or not. The majority of participants 73% (n=219) did not notice the state of the syringe used on them.

79% of respondents reported that television is the community source for health information, followed by community gatherings (19%); newspapers, radio and text messages for 2% of respondents.

### End of project study findings

After concluding all intervention activities and giving a period of one month’s grace, an end of project study was conducted between 26^th^ December 2011 and 8^th^ January 2012. The sample size was set at 300. The questionnaire for the end of project study was a combination of baseline and some additional variables. The end of project study was conducted in three *talukas* i.e. Tando Allahyar, Jhando Mari and Chamber (the same talukas as the intervention).

A total of 241 (80.3%) interviews were conducted in the community and 59 (19.7%) were exit interviews with patients attending the local clinics. Male and female respondents were 58% (n=174), and 42% (n=126) respectively. The mean age of study participants was 30.4± 9.5 years.

Compared to the baseline where 38% reported that the convenience/proximity was the main reason to select a healthcare provider in the case of illness, after the interventions 55% (n=165) participants responded that they would select a provider based no his/her qualifications followed by experience 38% (n=114) and convenience 6% (n=18).

In the baseline study only 9% thought that unsafe injections could cause hepatitis B and C in the community while in the post- intervention survey 78% respondents said that they thought the use of used syringes could cause hepatitis B and C.

**Figure 1:**
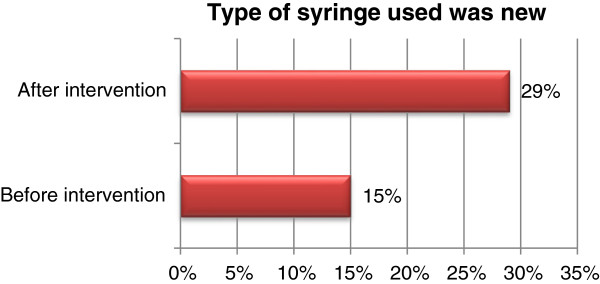
Providing details of patients’ knowledge about the type of syringe used for last injection.

In the baseline study 15% (n=45) respondents reported that they received a new syringe for injection use for their most recent injection. The post-intervention findings suggest an increase to 29% (n=87) (almost double). Figure 1 provides details.

The study participants were asked if they would request a new syringe if they had to have an injection in the future and if so what would be the reason for this. The study findings suggest that the majority 77% (n=231) thought they would ask for a new syringe while 15% (46) gave a negative reply and 8% (23) respondent said they did not know. 19% (n=57) said that the reason they would ask for a new syringe was to prevent them getting ‘diseases including hepatitis’.

The study participants were asked about a common belief prevailing in Pakistan which is “a drip (infusion) to gain strength”. The majority 76% (n=228) reported that ‘nutritious food’ provides strength while 8% (n=23) suggested that drips are necessary and 16% (n=48) reported that an injection for gaining strength is necessary.

Less than half 42% (n=126) of the participants said that they would question the provider about oral alternatives when a drip (IV infusion) is prescribed for them whereas 32% (n=96) said that they would immediately act on the prescriber’s advice (whatever is prescribed) and 26% (n=78) said that they would rather not have an injection or a drip.

The majority 95% (n=285) of respondents were aware that informative activities about injection safety were organized and held in the district.

## Discussion

Although the long-term impact of the intervention is hard to assess because of the short duration, there were some positive indicators. There was a two-fold increase in the perception of the community when selecting a healthcare provider showing that the qualification of provider rather than convenience is necessary. There was a significant (from 9% to 78%) increase in knowledge about the transmission dynamics of hepatitis B and C. There was also a significant increase (from 15% to 29%) in awareness about the state of the syringe used for the previous injection received.

Community-based interventions use a broad array of strategies that include education/behaviour change, engineering/technology, and legislation/enforcement. Educational strategies increase the awareness of injury risk or the importance of risk-reducing behaviours, and they may include media broadcasts, public service announcements, classroom instruction, or written material [20]. Our intervention also focused on the community and used different strategies. Our rationale to focus more on the community was that the healthcare providers have an ulterior motive to prescribe injections and working with the providers may not produce any positive change in improving irrational and unsafe use of injections. Working with the community and empowering the community to question the need for an injection and whether the syringe was new can have a meaningful impact in improving injection safety in Pakistan.

Community-based interventions have shown a positive impact in different parts of the world. A study in Guatemala between April 1997 and May 1998 evaluated the effectiveness of a set of information, education, and communication (IEC) strategies designed to increase the awareness of danger signs in pregnancy, delivery, or the postpartum period among pregnant or recently pregnant women. Among women using health clinics, the likelihood of having heard of danger signs nearly tripled between 1997 and 1998, when the clinic interventions were fully implemented. In 1999, those who had heard radio messages or participated in women's groups were, respectively, 3 times and 5 times more likely to have heard of danger signs in pregnancy [21]. A pilot project in two districts in Rwanda aimed to increase use of “Sûr'Eau”, a chlorine solution for drinking water treatment, through a partnership between community-based health insurance schemes and community health workers who promoted and distributed the product. Evaluation of the pilot, drawing on a difference-in-differences design and data from pre- and post-pilot household surveys of 4,780 households, showed that after 18 months of pilot implementation, knowledge and the use of the product increased significantly in the two pilot districts, but remained unchanged in a control district. The pilot was associated with a 40–42 percentage point increase in overall use [22].

Our study has shown multiple positive signs. In the baseline only 9% of community participants could say that hepatitis B and C can be transmitted due to unsafe injections. The knowledge level increased to 78% in the end of project report. And awareness about the state of the syringe used for the most recent injection doubled from 15% to 29% post-intervention.

The duration needed for such intervention is another area that needs attention. While there are no clear guidelines on the duration but the “Preventing Falls: A CDC Compendium of Effective Community-based Interventions from Around the World 2008” compiled by Stevens and Sogolow looked at 14 studies conducted around the world to prevent falls among elderly. The duration of interventions varied and ranged from four weeks to two years. We believe that if our intervention had continued for 18 months the level of knowledge retention and the overall impact could have been more far reaching and sustained.

There are certain limitations to the study. First and foremost, the short duration. It is hard to assess the actual impact on practices in this time period. A randomized control trial is the ideal design for an intervention but considering the complex nature of the issue of unsafe injection practices and the large scale of the problem, relatively simple interventions should be tried and improved based on lessons learned. The total cost of this project was $50,000 and can be increased and sustained with much better and lasting results in terms of improving knowledge and behaviour.

## Conclusion

The duration of intervention is the key besides other factors in producing a lasting impact. To improve injection safety in Pakistan it is necessary to have legislation which specifically addresses the rational prescription of injections and more importantly quackery. In order to address the issue of reuse of syringes, the introduction of reuse prevention devices (RUPs) for therapeutic injections and the sustained use of auto disable syringes for vaccination injections is strongly recommended.

## Competing interest

The authors declare that they have no competing interests.

## Authors’ contributions

AA conceptualized and wrote the proposal. He also supervised most of the activities in the field. SAS provided technical assistance and contributed to the proposal and manuscript development. KS contributed to the literature search. SK contributed to the manuscript development. All authors read approved and revised the final draft.
